# Neuroinflammation as a result of non‐neurotropic herpesvirus infection

**DOI:** 10.1111/imcb.70108

**Published:** 2026-03-31

**Authors:** Christian Münz

**Affiliations:** ^1^ Viral Immunobiology, Institute of Experimental Immunology University of Zurich Zurich Switzerland

**Keywords:** atypical memory B cells (ABCs), Epstein Barr virus (EBV), molecular mimicry, multiple sclerosis (MS), systemic lupus erythematosus (SLE)

## Abstract

The non‐neurotropic Epstein Barr virus (EBV) has been suggested to initiate the prodromal phase of multiple sclerosis (MS), often years before the first clinical symptoms. This review discusses mechanisms by which EBV might cause neuroinflammatory B‐cell migration to the central nervous system (CNS), as observed during primary CNS lymphomas and MS. Furthermore, mechanisms of molecular mimicry and autoreactive B‐cell expansion by EBV will also be summarized. Finally, approaches to target EBV‐mediated neuroinflammation for therapeutic interventions in people with MS (pwMS) will be explored. The recently provided information on EBV's association with MS gives exciting insights into the initiation of this autoimmune disease. Successful therapeutic interventions on the basis of this knowledge might provide evidence that EBV contributes also to the clinical phase of this autoimmune disease.

## Graphical abstract

Atypical memory B cells (ABCs) might stimulate autoreactive T cells to cause neuroinflammation and CNS myeloid cell activation during MS.

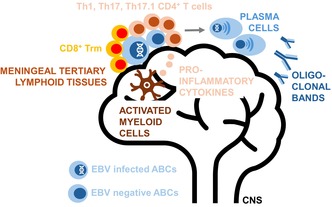

## INTRODUCTION

The central nervous system (CNS) needs to strike a delicate balance between immune control of neurotropic viruses and avoidance of immune pathology, especially immune mediated oxidative stress.^1^, ^2^ Some infectious challenges of the CNS are herpesviruses. In particular, the α‐herpesviruses, namely herpes simplex (HSV) and varicella zoster (VZV) virus with their known neurotropism, challenge the CNS during both acute infection, such as HSV encephalitis,^3^ and chronic infection, possibly explaining the beneficial effect of therapeutic VZV vaccination against dementia.^4^

However, in addition to the three α‐herpesviruses (HSV1, HSV2 and VZV), human herpesviruses contain six more members of the β‐ and γ‐herpesvirus subclass (cytomegalovirus or CMV, human herpesviruses 6A, 6B and 7, Epstein Barr virus or EBV, and Kaposi sarcoma‐associated herpesvirus or KSHV).^5^ These have at least part of their life cycle in hematopoietic cells (monocytes for CMV, T cells for HHV6A/6B/7, and B cells for EBV and KSHV). These hematopoietic host cells might carry the respective herpesviruses into the CNS during neuroinflammation and thereby contribute to CNS pathogenesis. In this review, I will primarily focus on EBV and its association with multiple sclerosis (MS), a demyelinating autoimmune disease of the CNS, in order to discuss the current hypotheses by which lymphotropic EBV might cause neuroinflammation.

EBV is a γ_1_‐herpesviruses with a double‐stranded DNA genome of around 170 kb.^6^ It is transmitted via saliva exchange between individuals and then most likely transcytosed across the mucosal epithelium to infect B cells of submucosal secondary lymphoid tissues, such as the tonsils.^7^, ^8^ In these B cells, EBV initially establishes a latent infection,^9^ replicating with the help of eight latent EBV proteins (6 nuclear antigens or EBNAs, and 2 latent membrane proteins or LMPs) and nontranslated small and micro RNAs through the proliferation of infected B cells.^6^ Only after chromatization of its genomes that circularize to episomes upon infection, EBV can initiate lytic replication to produce infectious virus particles.^10^ Latent and lytic EBV replication are linked to physiological differentiation stages of human B cells.^11^ All latent EBV protein expression can be found in naïve B cells of healthy virus carriers (latency III).^12^ EBNA1, LMP1 and LMP2 expression (latency II) can be observed in B cells with a germinal center phenotype.^12^ Only EBNA1 expression (latency I) is observed in homeostatically proliferating memory B cells^13^ that shut down all EBV protein expression as resting memory B cells.^12^, ^14^ From this memory, B‐cell reservoir of persistent EBV infection EBV can reactivate into lytic replication upon plasma cell differentiation.^15^ At mucosal surfaces, this is thought to lead to shedding into saliva for transmission. With this B‐cell‐adapted life cycle, EBV is among the most successful pathogens in humans, establishing persistent infection in more than 95% of the adult human population.^16^ This prevalence is completed within the first years of life in developing countries, such as in sub‐Saharan Africa,^17^ while primary infection can be delayed into the second decade of life in around one third of individuals in developed countries such as the United States, and is then more frequently associated with infectious mononucleosis (IM), an overshooting expansion of EBV specific CD8^+^ T cells.^18^ Thus, EBV influences both B‐cell differentiation and strong T‐cell activation, challenging tolerance mechanisms and immune suppressive mechanisms that prevent neuroinflammation.

## TUMORIGENESIS BY EPSTEIN BARR VIRUS INFECTION

One consequence of EBV adapting its gene expression to B‐cell differentiation stages and also driving B cells for memory B‐cell differentiation through these differentiation stages is that EBV‐positive B‐cell lymphomas develop from these differentiation stages in a small subset of virus carriers.^19^ Latency III can immortalize human B cells into lymphoblastoid cell lines (LCLs) in vitro and is found in a subset of diffuse large B‐cell lymphomas (DLBCL), as well as in B‐cell lymphoproliferations of immune compromised hosts, such as in post‐transplant lymphoproliferative disease (PTLD). For all malignancies with more restricted EBV latent antigen expression, such as latency II in Hodgkin lymphoma and latency I in Burkitt lymphoma, compensatory host genome mutations or co‐infections replace EBV gene products, such as c‐myc translocation into one of the immunoglobulin loci of Burkitt lymphoma, compensating for EBNA2 driven c‐myc upregulation,^20^, ^21^ and KSHV co‐infection in primary effusion lymphoma (PEL) with EBV latency I.^22^ The respective tumors preserve phenotypes of EBV infected B‐cell differentiation stages with Hodgkin and Burkitt lymphomas originating from germinal center B cells and probably also acquiring host cell mutations through the germinal center somatic hypermutation machinery.^23^ Thus, the association of different EBV infection programs with B‐cell differentiation stages is also to a large extent conserved in EBV‐associated B‐cell lymphomas.

The homing capacity of B‐cell differentiation stages to distinct tissues is also conserved in some of these EBV‐associated lymphomas. For example, primary CNS lymphomas (PCNSL) in immune compromised conditions such as human immunodeficiency virus (HIV) coinfection are mostly EBV‐positive.^24^ While EBV‐negative PCNSL are primarily of the activated B‐cell subtype of DLBCLs (ABC‐DLBCL) and enriched for the IGHV4‐34 variable domain of the B‐cell receptor (BCR), EBV‐positive PCNSL carry few host genome mutations and instead of the immune escape by MHC loss that is often found in EBV‐negative PCNSL, EBV‐positive PCNSL seem to induce an immune suppressive tumor microenvironment by inhibitory receptor stimulation, such as PD‐1, LAG‐3 and TIM‐3.^25^ During immune check‐point blockade, blocking PD‐1 with antibodies, encephalitis was recorded as an adverse therapy event in a subset of melanoma patients.^26^ In a small number of these, EBV specific CD4^+^ T‐cell infiltrates and EBV infected B cells were found postmortem in their CNS. Similarly, PD‐1 blocking during EBV infection of mice with reconstituted human immune system components (humanized mice) allows EBV to replicate to higher viral loads^27^, ^28^ followed by migration of EBV‐infected cells to the CNS.^28^ Thus, diminished EBV‐specific immune control seems to allow EBV‐positive DLBCLs, possibly mainly of a nongerminal center B‐cell phenotype to migrate to the CNS. Interestingly, some of these PCNSL characteristics we will re‐encounter for B cells that infiltrate the CNS during MS.

## ASSOCIATION OF EPSTEIN BARR VIRUS INFECTION WITH AUTOIMMUNITY

EBV viral loads are elevated in several autoimmune diseases such as a 10‐fold increase in rheumatoid arthritis (RA) and a 40‐fold increase in systemic lupus erythematosus (SLE),^29^, ^30^ but not multiple sclerosis (MS).^31^, ^32^ Furthermore, EBV‐specific antibodies are elevated in RA, SLE and MS.^33^, ^34^ Finally, spontaneous EBV transformed B‐cell outgrowth was more readily observed in MS and RA but not SLE.^35^, ^36^ For several decades it remained, however, unclear whether dysregulated EBV infection and immune responses to it were just secondary to autoantigen mediated B‐cell stimulation with possibly elevated lytic EBV replication upon plasma cell differentiation, or whether they preceded and therefore contributed to autoimmunity. In order to address this chicken and egg problem, a longitudinal study in the American military personnel was conducted to investigate the prodromal phase of the disease in more than 900 service members that developed MS.^37^ It was determined that all except for one of these people with MS (pwMS) became EBV seropositive on average seven and a half years prior to first clinical MS symptoms. From time of seroconversion neurofilament light chain (NfL) as a serum marker of neuroaxonal damage and elevated EBV‐specific antibodies were found in those EBV‐infected individuals that then went on to develop MS. Antibodies against 200 other human viruses were not altered in the prodromal phase of MS‐ and CMV‐specific antibodies correlated even with a slight protection (OR 0.7).^37^, ^38^ The authors calculated a more than 30‐fold increased risk to develop MS after EBV infection,^37^ compared to a threefold elevated risk mediated by the main genetic risk factor, the MHC class II molecules HLA‐DRB1*1501, for the disease.^38^ This suggested that EBV infection is a necessary but not sufficient requirement for the development of MS. However, the mechanism of this association remains to be fully elucidated.

Three main and not mutually exclusive hypotheses are currently discussed, namely molecular mimicry between EBV and CNS autoantigens, defective EBV‐specific immune control that allows infected B cells to migrate to the CNS and stimulate pathogenic T cells, and EBV infection immortalizing autoreactive B cells that then stimulate autoreactive T cells and produce autoantibodies (Table 1). For each of these, experimental evidence has been provided. EBNA1‐specific antibodies have been found to cross‐react with myelin basic protein (MBP),^39^ GlialCAM,^40^ anoctamin 2 (Ano2)^41^ and α‐crystallin B (CRYAB).^42^ Cross‐reactive T‐cell responses have also been identified for EBNA1 with MBP, anoctamin 2 and α‐crystallin B.^32^, ^42^, ^43^, ^44^ These studies argue that during EBV infection CNS autoantigen‐specific B‐ and T‐cell responses are primed to EBNA1 that then present CNS autoantigens to autoreactive and cross‐reactive CD4^+^ T cells. Since all of the cross‐reactive antibody targeted epitopes are intracellular, the respective BCRs and antibodies probably play mainly a role in antigen processing for T‐cell stimulation. This might occur more often during poorly controlled EBV infection, such as during IM, but also immune systems carrying the main genetic risk factor for MS have been shown to control EBV less well in humanized mice.^45^ This leads to elevated EBNA1 specific antibody titers in humanized mice^45^ that have also been observed in HLA‐DRB1*1501 positive individuals.^46^ Therefore, genetic predisposition to MS might allow EBV to replicate to higher viral loads and thereby expand CNS homing B cell subpopulations.^47^

**Table 1:** Possible mechanisms by which EBV infection initiates MS.

No.	Title	Explanation	Antigen	Effector function	References
1	Molecular mimicry	Cross‐reactivity between EBV and CNS autoantigens	EBNA1, MBP, Ano2, GlialCam, CRYAB	Cross‐reactive pro‐inflammatory T cell responses	[39, 40, 41, 42, 43, 44]
2	EBV infected ABC migration to the CNS	EBV infection enforces the ABC program of B cells that allows migration to the CNS and then recruitment of T cells	EBV antigens	Attraction and stimulation of EBV specific pro‐inflammatory T cell responses	[47, 48, 49]
3	EBV infected autoreactive B cell expansion	EBV infects precursors of autoreactive B cells, resulting in their expansion and affinity maturation	Autoantigens	Stimulation of autoreactive T cell responses and production of autoantibodies	[56, 58, 59]

EBV infection indeed expands CXCR3^+^T‐bet^+^ atypical memory B cells (ABCs) in humanized mice that then infiltrate the brain.^47^ Similarly, ABCs have been found to constitute the majority of B cells in postmortem brains of pwMS^48^ and EBV‐infected B cells have been found in postmortem brains and draining cervical lymph nodes of pwMS.^49^, ^50^, ^51^ VLA‐4 and CXCR3 allow ABC migration to the CNS.^47^, ^48^ Accordingly, EBV infected B cells very efficiently migrate across endothelial barriers.^52^ These EBV‐infected ABCs then attract T cells by chemokines that are recognized by CCR5 and CXCR3, and the infiltrating activated CD4^+^ T cells correlate with NfL release as a sign of neuroaxonal damage.^47^ Interestingly, some of the EBV‐expanded CNS homing ABC clones also express the IGHV4‐34 variable domain of the BCR that is found on PCNSL. In addition, these ABCs rapidly differentiate into antibody producing plasma cells and could be the source of the MS defining oligoclonal bands in the cerebrospinal fluid (CSF).^48^ Moreover, they are efficient stimulators of T‐cell responses.^53^ This could also be enhanced by EBV, possibly mainly by a specific EBNA2 allele (EBNA2 1.2), mediated transcription of genetic MS risk loci that mainly affect antigen presenting cell interaction with T cells.^54^, ^55^ Thus, expansion of CNS homing ABCs during delayed primary EBV infection could allow these cells to home to the CNS to start neuroinflammation after attraction of T cells.

As a third mechanism, it has been proposed that EBV infection expands autoimmune B cell populations. During symptomatic primary EBV infection, namely IM, which predisposes individuals to MS with a twofold increased risk, α‐crystallin B specific antibodies cross‐reacting with EBNA1 have been observed.^56^ A transient development of autoantibodies during IM was also detected earlier for other autoantigens, such as myelin oligodendrocyte glycoprotein (MOG) in a subset of IM patients.^57^ Furthermore, during SLE it was recently reported that EBV infected T‐bet^+^ B cells carry B‐cell receptors (BCRs) that recognized nuclear antigens and might serve as precursors of antinuclear antibody (ANA) producing plasma cells.^58^ This finding of EBV infection in autoreactive BCR carrying CD27^+^CD21^low^ and partially CD11c and CXCR3 expressing B cells was recently also extended to MS patients.^59^ Some BCRs of these at least in part EBV infected CXCR3^+^ B cells recognized EBNA1 with cross‐reactivities to Ano2 and for one clone CRYAB. Thus, EBV mediated expansion of ABCs might generate autoantigen‐specific B‐cell clones that serve as efficient antigen presenting cells to stimulate autoreactive T‐cell responses. A combination of these three mechanisms might explain why autoimmunity is fortunately a rare complication of EBV infection. Namely, EBV infection expands tissue homing B cells but only if these carry BCRs that recognize autoantigens in these tissues autoimmunity might ensue.

## IMMUNE CONTROL OF EPSTEIN BARR VIRUS

How might these EBV‐infected tissue homing B cell reservoirs be controlled? This could indeed pose quite a challenge because EBV infection is thought to be primarily controlled by early differentiated cytotoxic CD8^+^ T cells with a significant expansion capacity.^6^, ^60^ The mutations underlying primary immunodeficiencies (PID) or inborn errors of immunity (IEI) affect genes that are required for the development, expansion, stimulation and cytotoxicity of lymphocytes, such as CTPS, 4‐1BB, CD27, IL‐27 and perforin. Indeed, healthy EBV carriers have primarily CD27^+^ early differentiated EBV‐specific effector memory CD8^+^ T cells in their peripheral blood (Tem1), while CMV specific CD8^+^ T cells can be primarily found in the terminally differentiated CD45RA re‐expressing effector memory compartment (Temra).^61^, ^62^ Such early differentiated EBV‐specific CD8^+^ T cells persist also better after adoptive transfer into preclinical animal models.^63^ This early differentiated CD8^+^ T‐cell phenotype seems essential for continuous EBV‐specific immune control, as exemplified by CD27 and its ligand CD70 deficiencies, that result in hyperinflammatory (hemophagocytic lymphohistiocytosis) and Hodgkin disease after primary EBV infection in the majority of affected individuals.^64^ Similarly, in humanized mice, CD27 blocking with antibodies leads to increased viral loads and lymphomagenesis after EBV infection.^65^ Such loss of immune control is not sufficient to cause MS‐like symptoms in humanized mice, even so PD‐1 blocking that also increases viral loads was reported earlier to allow EBV infection to gain access to the CNS.^26^, ^28^ Autoantigen recognition by the BCRs of CNS homing EBV infected B cells might be required to initiate prodromal MS pathology. Particularly, lytic EBV antigen specific CD8^+^ T cells seem to need CD27 to efficiently expand during lytic virus reactivation. Indeed, the highest frequencies of EBV specific CD8^+^ T cells seem to recognize early lytic EBV antigens^66^ and some of these specificities can make up 40% of the CD8^+^ T cell pool during IM.^67^ Among the latent EBV antigens, EBNA3A, EBNA3B, EBNA3C and LMP2 are the most frequently CD8^+^ T‐cell targets.^66^ Accordingly, T cells expanded with autologous LCLs have been used for adoptive transfer therapies of EBV lymphomas, mainly PTLD^68^ and later these have been focused on LMP2‐specific T cells.^69^, ^70^ However, it was realized that these adoptively transferred CD8^+^ T cells most likely survive better and maintain their effector function in the presence of CD4^+^ T cells.^71^, ^72^ Therefore, EBV antigens for CD4^+^ T‐cell responses were explored. EBNA1 was found to be consistently recognized by CD4^+^ T cells of healthy virus carriers.^73^, ^74^ Even so EBNA1 specific T cell lines also demonstrated clinical efficacy against PTLD,^75^, ^76^ EBNA1 was later paired with LMPs for T‐cell expansion for adoptive transfer into patients with EBV associated malignancies.^77^ EBNA1 plus LMP2 was then also incorporated into vaccines to induce such protective T cell responses,^78^, ^79^, ^80^, ^81^ which were however so far not further pursued than in preclinical models and phase I clinical trials. Nevertheless, CD27^+^ early differentiated EBNA1 and LMP2 specific T cells might be efficient in controlling EBV infection, targeting even latently infected B cells that only express EBNA1, such as in latency I Burkitt lymphoma.

## COMPROMISED EPSTEIN BARR VIRUS SPECIFIC IMMUNE CONTROL IN MS

In tissues, including the CNS, but also mucosal surfaces, however, terminally differentiated T cells accumulate that often take up residency at these sites.^82^, ^83^ This also applies to T cells in postmortem brains and CSF of pwMS.^84^, ^85^ Furthermore, LCL reactive and likely EBV specific T cells have been found among the expanded clones in CSF of pwMS.^86^, ^87^ Tissue resident T cells make up the majority of CD8^+^ T cells in the nasal associated lymphoid tissue (NALT) of humanized mice and tonsils of healthy children.^88^ In the NALT, they are less able to control EBV infection than CD27^+^ CD8^+^ T cells in the spleen of humanized mice. These findings are consistent with CD27 being required for EBV specific immune control by CD8^+^ T cells as discussed above. This might also explain the delayed control of mucosal EBV shedding compared to blood viral loads in IM patients.^89^ Analogously, less efficient immune control in the CNS by tissue resident T cells might allow for the development of an EBV infected B‐cell reservoir that leads to PCNSL in HIV‐infected individuals and drives elevated EBNA1 antibody titers in pwMS.^24^, ^90^ This reservoir could nucleate the tertiary lymphoid structures that have been found associated with MS severity.^91^ Controlling this tissue reservoir that might initiate and possibly also continue to promote MS pathology might be challenging.

Indeed, trials with adoptively transferred EBNA1 and LMP specific T cells into pwMS did give mixed results. An initial phase I clinical trial demonstrated improvement by reduced CNS lesions in secondary progressive MS, following in most pwMS a relapsing remitting phase (RR‐MS) of the disease and currently without treatment options.^92^ However, a phase I/II clinical trial (NCT03283826) by Atara Biotherapeutics was terminated because it did not reach its primary endpoint, namely disability improvement which is, however, difficult to achieve due to already quite significant neuronal damage in secondary progressive MS. Another possibility to target such EBV infected B cells that infiltrate the CNS and/or give rise to autoantibody producing plasma cells are possibly natural killer (NK) cell responses.^93^ It was proposed that adaptive NKG2C^+^ NK cells that are expanded by CMV infection might recognize in an HLA‐E and LMP1 allele specific fashion pathogenic B cell populations. Which exact EBV infected B cell differentiation stage is targeted by these B cells and if the protective NK cells can and need to cross the blood–brain‐barrier (BBB) requires further studies. Such a protective effect by CMV‐expanded NK cells would however explain the decreased risk for MS in CMV infected individuals.^38^ Similar to possible difficulties of these lymphocyte populations to cross the BBB, CD20 specific antibody, such as Rituximab and Ocrelizumab, mediated B cell depleting therapies that are currently used for MS treatment primarily abolish relapses during RR‐MS^94^, ^95^ and presumably hardly reach the CNS reservoir of B cells that might drive smoldering MS, also referred to as progression without relapse activity (PIRA). Therefore, emboldened by the success of CD19 specific CAR T‐cell therapies in SLE,^96^, ^97^ similarly deep tissue‐B cell depleting therapies might also be tested in MS. Indeed, ClinicalTrials.gov lists more than 10 clinical trials for this purpose. Therefore, it will be interesting if this decreases PIRA and possibly also depletes a pathogenic EBV‐infected B‐cell reservoir in MS that leads to elevated EBNA1‐specific antibody titers. Success in these trials would also provide an opportunity to more selectively target CNS homing B‐cell subsets or improve on EBV‐specific T‐cell lines to reflect more the protective T‐cell phenotype of healthy EBV carriers for transfer in pwMS.

## CONCLUSIONS

**Figure 1:**
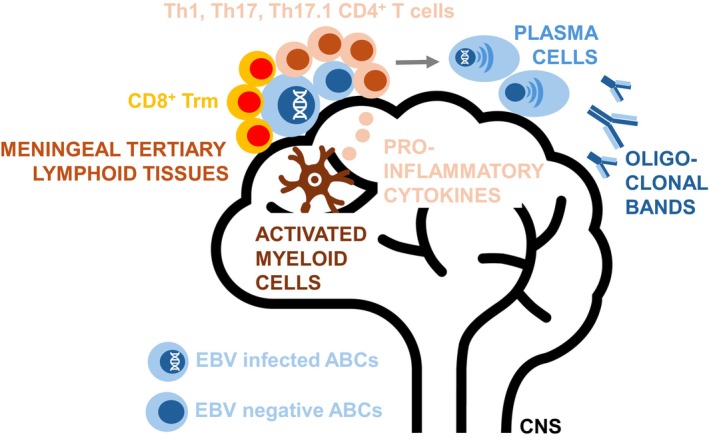
Submeningeal lymphocyte aggregates. CXCR3^+^T‐bet^+^ atypical memory B cells (ABCs) might stimulate autoreactive Th1, Th17 and Th17.1 CD4^+^ T cells to cause neuroinflammation and CNS myeloid cell activation during MS. This could occur in submeningeal B‐ and T‐cell aggregates and might also lead to ABC differentiation into plasma cells that then produce oligoclonal bands. CD8^+^ T cells might have a reduced capacity to restrict EBV‐infected ABCs at these sites due to their phenotype of terminally differentiated tissue resident cells.

Epidemiological studies on the prodromal phase of MS have now firmly established that EBV infection is required but not sufficient to initiate MS. The ability of EBV to clonally expand CNS homing B‐cell populations might unfortunately affect autoreactive or EBV and autoantigen cross‐reactive B cells that, once in the CNS, differentiate to autoantibody producing plasma cells and attract as well as stimulate autoreactive T‐cell responses. During RR‐MS, these pathogenic B‐ and T‐cell clusters might be periodically enlarged by CNS infiltrating lymphocytes, while the progressive stage of the disease (PIRA during RR‐MS treatment and progressive MS) might represent B‐ and T‐cell cluster reactivity that are resident in the CNS (Figure 1). Future clinical trials will demonstrate if these can be reached by EBV‐specific, B‐cell subset‐ or generally B cell depleting CAR T cell therapies. They might also be affected by prophylactic vaccination and antivirals that are currently tested against EBV.^6^, ^98^, ^99^ Nevertheless, we have now one or possibly several working models how non‐neurotropic EBV might cause neuroinflammation, which we can further investigate and also use as guidance to develop additional therapies for pwMS.

## ETHICS STATEMENT

No animal experiments or human materials were used for this review. Therefore, no ethics approvals were required.

## AUTHOR CONTRIBUTIONS

CM wrote and edited this manuscript. CM also developed the graphics.

## CONFLICT OF INTEREST

The author declares no known competing financial interests or personal relationships that could influence this work.

## Data Availability

Data sharing is not applicable to this article, as no datasets were generated or analyzed during the current study.
